# The ghost and the machine

**DOI:** 10.1186/2041-2223-1-11

**Published:** 2010-11-02

**Authors:** Mark A Jobling

**Affiliations:** 1 Department of Genetics, University of Leicester, Leicester, UK

## 

In Monty Python's 'Four Yorkshiremen' sketch [1], the tuxedo-clad, cigar-puffing gents sit quaffing wine, and get to reminiscing. The conversation moves round the group:

"Who'd have thought thirty year ago we'd all be sitting here drinking Château de Chasselas, eh?"

"In them days we was glad to have the price of a cup o' tea."

"A cup o' cold tea."

"Without milk or sugar."

"Or tea."

"In a cracked cup, an' all."

"Oh, *we *never had a cup. We used to have to drink out of a rolled up newspaper."

And so it goes on, the competitive reminiscences becoming more and more absurd in their invocation of hardship.

Listening to geneticists of a certain age is sometimes a bit like this. Some event or remark sets off the litany: complaints from the lab about the slowness of the central sequencing service are met with harrumphing, and a tale about polyacrylamide gels and ^35^S labelling; this leads to a diatribe about the intricacies of cDNA library construction; at some point the 'three waterbaths' story of the early days of PCR is wheeled out; an esteemed colleague might raise the stakes by recounting the tribulations of making his own restriction enzymes.

Is this just the universal nostalgia for a past era that is one of the hallmarks of ageing? To an extent, yes, but there's more to it than that. Certainly in the area of scientific endeavour within which many readers of this journal work, the average project has become experimentally far less interesting and challenging that it used to be.

During my own PhD I grew cell-lines, made YAC, cosmid and phage libraries, did pulsed-field gel mapping, southern blotted, subcloned, and enjoyed plentiful exposure to phenol and radioisotopes, as did my lab-mates; nowadays my students spend their time putting *Taq *polymerase through its paces, waiting for the garish purple DNA extraction robot to beep, ordering kits, and outsourcing the really tricky stuff to the cheapest supplier. The benches can sometimes become repositories of paper - dusty adjuncts to the desk and computer - but at least the occasional pipette is in operation. My genome centre colleagues have been presented with spacious and gleaming labs, but every time I visit they lie empty, the workers packed instead into a rather-too-snug office.

Part of the unease about this change is to do with the gradual abandonment of manual tasks that are difficult and varied and satisfying. In Matthew Crawford's book *The Case for Working with Your Hands, or Why Office Work is bad for us *[2], he extols the virtues of manual and mechanical competence, personifying his argument by having moved from a PhD in political philosophy from Chicago to run his own independent motorcycle repair shop. OK - his kind of manual work, fixing broken motorbikes, is not exactly molecular genetics, but there are nonetheless interesting parallels. Crawford bemoans the move by manufacturers towards more complex products that are more difficult to tinker with, making users 'more passive and more dependent', and the declining opportunity for 'the kind of spiritedness that is called forth when we take things in hand for ourselves'. Any ageing geneticist who signs an order for some new and expensive kit knows exactly what he means.

The thing that today's PhD students are doing that their supervisors didn't do much of is computing, and, for some of the time at least, bioinformatics - what the wrench-wielding Crawford would class as 'the most ghostly kind of work'. Here, the thinking part is just as interesting and challenging as ever it was, and maybe even more so. But the manual, mechanical stuff has been done somewhere else, by someone else. The power of whole genome and exome sequences, high-throughput genotyping data, population genomics and the like is (to use an oft-abused word) truly awesome, as is nicely illustrated by a recent review issue of *Human Molecular Genetics *[3]. And yet, an appreciation of how data are generated, and some degree of intimacy with the sources of experimental error, seem crucial to producing a biologically grounded scientist. We see this in the value of practical classes for undergraduates in getting them to grasp biological principles and solve biological problems.

Ideally, the bioinformatician and the experimental scientist would be combined in one supercapable individual, but producing students who can be both ghosts and mechanics is a difficult challenge. The best we can currently do is to combine them in multidisciplinary teams, but even here it is hard to recruit a bioinformatician into a laboratory science group - they seem more inclined to stick to their own kind, in environments without the inconvenience and mess of actual data generation.

Like the four Yorkshiremen, in our PhD days we were poor but we were happy. Now the young are the idle data megarich, and we worry about them.
